# Blood temperature and perfusion to exercising and non‐exercising human limbs

**DOI:** 10.1113/EP085383

**Published:** 2015-09-13

**Authors:** José González‐Alonso, José A. L. Calbet, Robert Boushel, Jørn W. Helge, Hans Søndergaard, Thor Munch‐Andersen, Gerrit van Hall, Stefan P. Mortensen, Niels H. Secher

**Affiliations:** ^1^ Centre for Sports Medicine and Human PerformanceBrunel University LondonUxbridgeUK; ^2^ The Copenhagen Muscle Research CentreRigshospitaletUniversity of CopenhagenCopenhagenDenmark; ^3^ Department of Physical Education, and Research Institute of Biomedical and Health SciencesUniversity of Las Palmas de Gran CanariaLas Palmas de Gran CanariaSpain; ^4^ School of KinesiologyUniversity of British ColumbiaVancouverCanada; ^5^ Centre for Healthy AgeingDepartment of Biomedical SciencesUniversity of CopenhagenCopenhagenDenmark; ^6^ Department of Cardiovascular and Renal ResearchUniversity of Southern DenmarkOdenseDenmark; ^7^ Department of AnaesthesiaRigshospitaletUniversity of CopenhagenDenmark

## Abstract

**New Findings:**

- **What is the central question of this study?**
  Temperature‐sensitive mechanisms are thought to contribute to blood‐flow regulation, but the relationship between exercising and non‐exercising limb perfusion and blood temperature is not established.
- **What is the main finding and its importance?**
  The close coupling among perfusion, blood temperature and aerobic metabolism in exercising and non‐exercising extremities across different exercise modalities and activity levels and the tight association between limb vasodilatation and increases in plasma ATP suggest that both temperature‐ and metabolism‐sensitive mechanisms are important for the control of human limb perfusion, possibly by activating ATP release from the erythrocytes.

Temperature‐sensitive mechanisms may contribute to blood‐flow regulation, but the influence of temperature on perfusion to exercising and non‐exercising human limbs is not established. Blood temperature (*T*_B_), blood flow and oxygen uptake (${\dot{V}}_{O_{2}})$ in the legs and arms were measured in 16 healthy humans during 90 min of leg and arm exercise and during exhaustive incremental leg or arm exercise. During prolonged exercise, leg blood flow (LBF) was fourfold higher than arm blood flow (ABF) in association with higher *T*_B_ and limb ${\dot{V}}_{O_{2}}.$ Leg and arm vascular conductance during exercise compared with rest was related closely to *T*_B_ (*r* ^2^ = 0.91; *P < *0.05), plasma ATP (*r* ^2^ = 0.94; *P* < 0.05) and limb ${\dot{V}}_{O_{2}}$ (*r* ^2^ = 0.99; *P < *0.05). During incremental leg exercise, LBF increased in association with elevations in *T*_B_ and limb ${\dot{V}}_{O_{2}},$ whereas ABF, arm *T*_B_ and ${\dot{V}}_{O_{2}}$ remained largely unchanged. During incremental arm exercise, both ABF and LBF increased in relationship to similar increases in ${\dot{V}}_{O_{2}}.$ In 12 trained males, increases in femoral *T*_B_ and LBF during incremental leg exercise were mirrored by similar pulmonary artery *T*_B_ and cardiac output dynamics, suggesting that processes in active limbs dominate central temperature and perfusion responses. The present data reveal a close coupling among perfusion, *T*_B_ and aerobic metabolism in exercising and non‐exercising extremities and a tight association between limb vasodilatation and increases in plasma ATP. These findings suggest that temperature and ${\dot{V}}_{O_{2}}$ contribute to the regulation of limb perfusion through control of intravascular ATP.

## Introduction

Local tissue and blood temperature (*T*_B_) increases with elevations in skeletal muscle metabolism and heat production during dynamic exercise (Sproule & Archer, 1959; Saltin & Hermansen, 1966; González‐Alonso *et al*. 1999, 2000). However, the influence of temperature on perfusion to exercising and non‐exercising human limbs is not established. In humans, the increases in local perfusion and oxygen uptake (${\dot{V}}_{O_{2}})$ during leg exercise are much greater than during arm exercise, reflecting differences in muscle mass and work capacity (Secher *et al*. 1977; Knight *et al*. 1992; Volianitis & Secher, 2002; Calbet *et al*. 2004; Mortensen *et al*. 2005). Limb tissue and blood temperatures depend upon the balance between heat production and endogenous heat transfer. In exercising limbs, heat is transferred from the working muscles to the neighbouring tissues and the overlying skin as well as to the body core. This is made possible via the flowing blood (convective heat transfer) and direct intercellular heat conduction (conductive heat transfer; Barcroft & Edholm, 1943; Pennes *et al*. 1948; González‐Alonso *et al*. 2000). Differences in ${\dot{V}}_{O_{2}}$ and thus metabolic heat production between the exercising lower and upper limbs could affect the increase in *T*_B_ and the relationship between *T*_B_ and perfusion in exercising and non‐exercising limbs if the differences in heat production are not matched by proportional changes in endogenous heat transfer. To date, no study has examined the relationships amongst limb *T*_B_, perfusion and aerobic metabolism during separate and combined lower and upper limb exercise to determine whether a coupling between *T*_B_ and limb perfusion is still apparent when accounting for differences in metabolism and heat production.

Understanding of thermoregulation during exercise is largely based on the regulation of skin blood flow and sweating in resting limbs (Johnson *et al*. 2014). Yet the local thermal stimuli modulating these key thermoregulatory responses during exercise are likely to be different in the exercising and non‐exercising limbs, unless increases in temperature in the exercising limbs lead to similar elevations in blood and tissue temperature in the non‐exercising limbs. The net heat transfer from the exercising limbs to the trunk and head results in increased core and brain temperatures (Saltin *et al*. 1966; Nybo *et al*. 2002; Kenny *et al*. 2003; Trangmar *et al*. 2014). In non‐exercising limbs during prolonged leg exercise, however, forearm venous *T*_B_ and muscle temperature are lower and do not increase to the same extent as core and active leg muscle and blood temperatures (González‐Alonso *et al*. 1999; Jay *et al*. 2007). To shed light on the mechanisms of temperature and limb blood‐flow regulation, it is timely to investigate the impact of haemodynamic and thermodynamic events in exercising limbs on central and non‐exercising limb perfusion and *T*_B_.

Temperature is one of a congregate of metabolic byproducts proposed to contribute to regulation of limb tissue perfusion (Barcroft & Edholm, 1943). In support of a role for hyperthermia, increases in local blood and muscle temperatures are associated with similar elevations in limb perfusion during both isolated leg and whole‐body heat stress (Pearson *et al*. 2011; Heinonen *et al*. 2011; Chiesa *et al*. 2015), irrespective of differences in systemic temperature and haemodynamic responses between conditions (Chiesa *et al*. 2015). Although small compared with exercise hyperaemia, this hyperthermia‐mediated limb hyperaemia is maintained during combined heat stress and one‐legged knee‐extensor exercise (Pearson *et al*. 2011; Chiesa *et al*. 2015). The thermal hyperaemia in resting limbs is associated only in part with metabolic vasodilatation, because the concomitant elevation in limb ${\dot{V}}_{O_{2}}$ is too small to account for the increase in perfusion (Pearson *et al*. 2011; Chiesa *et al*. 2015). Thus, hyperthermia induces vasodilatation of the limb tissue vascular beds through other mechanisms, which may be temperature sensitive. Along these lines, hyperthermia is associated with elevations in intravascular concentration of the potent vasoactive substance ATP (Pearson *et al*. 2011), accompanying skeletal muscle, skin and bone vasodilatation (Heinonen *et al*. 2011; Pearson *et al*. 2011). During exercise in normal environmental conditions, plasma ATP also increases in the forearm and leg circulations (Forrester & Lind, 1969; Forrester, 1972; González‐Alonso *et al*. 2002; Mortensen *et al*. 2011), possibly in response to alterations in erythrocyte oxygen binding and other adjuvant metabolic, thermal and mechanical stimuli (Bergfeld & Forrester, 1992; Ellsworth *et al*. 1995; Sprague *et al*. 1998; Wan *et al*. 2008; Kalsi & González‐Alonso, 2012). These observations raise the question of whether plasma ATP would increase during exercise with the lower and upper limbs in parallel to the increases in *T*_B_ and the reductions in blood oxygenation that accompany increases in skeletal muscle aerobic metabolism.

The main purpose of this study, therefore, was to investigate the relationships amongst perfusion, *T*_B_ and ${\dot{V}}_{O_{2}}$ in exercising and non‐exercising limbs during separate and combined upper and lower limb exercise. In addition, the relationship between *T*_B_ and plasma ATP concentration was examined during combined upper and lower limb exercise to shed light on the potential role of temperature‐ and metabolism‐sensitive intravascular ATP release in exercise hyperaemia. Lastly, the impact of *T*_B_ in the exercising limbs on central blood temperature was identified in a group of trained individuals. We hypothesized that limb perfusion, *T*_B_ and aerobic metabolism would be closely coupled in exercising and non‐exercising extremities, as would be plasma ATP and *T*_B_ and limb vasodilatation. Furthermore, the elevations in *T*_B_ and perfusion of the exercising limbs during incremental exercise would be associated with increases in *T*_B_ and blood flow in the central circulation, but not in the non‐exercising limbs.

## Methods

Twenty‐eight participants took part in two studies. The 16 healthy recreationally active subjects (10 males and six females) participating in study 1 possessed a mean (±SD) age of 31 ± 7 years, body mass of 73 ± 12 kg, height of 175 ± 8 cm and maximal oxygen uptake (${\dot{V}}_{O_{2}\max})$ of 3.4 ± 0.6 l min^−1^ (48 ± 6 ml kg^−1^ min^−1^).The age, body mass, height and ${\dot{V}}_{O_{2}\max}$ of the 12 endurance‐trained male cyclists included in study 2 were 27 ± 3 years, 76 ± 7 kg, 182 ± 10 cm and 4.7 ± 0.3 l min^−1^ (62 ± 3 ml kg^−1^ min^−1^), respectively. All participants were fully informed of any risks and discomforts associated with the experiments before giving their informed written consent to participate. These studies conformed to the Code of Ethics of the World Medical Association (*Declaration of Helsinki*) and were approved by the Ethics Committee of Copenhagen (KF01‐223/03) and the Capital Region of Denmark (H‐4‐2009‐097).

### Experimental protocols

**Figure 1:**
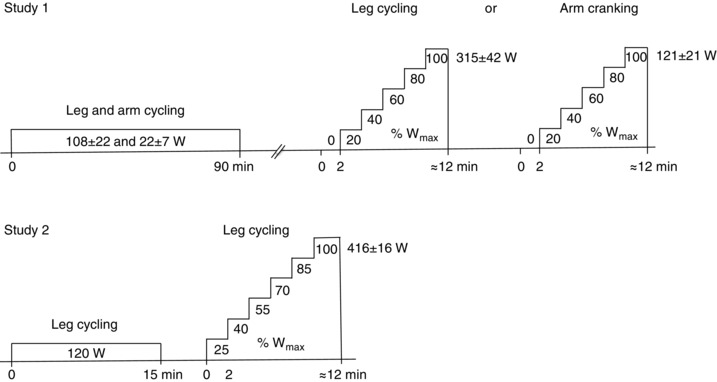
**Sequence of the experimental protocols** In study 1, participants first performed 90 min of submaximal combined leg‐ and arm‐cycling exercise followed by 1 h of rest and then completed two counterbalanced incremental cycling bouts to volitional exhaustion, one with their legs and one with their arms [maximal work rate (*W*_max_) 315 ± 42 and 121 ± 21 W, respectively]. The two incremental exercise bouts were separated by ∼30 min of rest and were started with unloaded pedalling, with the workload increasing every 2 min thereafter to elicit 20, 40, 60, 80 and 100% of the previously determined *W*_max_. In study 2, the participants completed an incremental leg‐cycling test to volitional exhaustion, with the workload increased every 2 min to elicit 25, 40, 55, 70, 85 and 100% of the previously determined *W*_max_ (416 ± 16 W).

In study 1, the participants were first tested at rest and during 90 min of submaximal leg‐ and arm‐cycling exercise (108 ± 22 and 22 ± 7 W, respectively, or 34 and 18% of the corresponding maximal work rate (*W*_max_), and then during incremental leg‐cycling or arm‐cranking exercise to volitional exhaustion (*W*_max_ 315 ± 42 and 121 ± 21 W, respectively), following 1 h of rest (Fig. 1). The two incremental cycling bouts were separated by ∼30 min of rest and were preceded by unloaded pedalling. The workload during the incremental tests was increased every 2 min to elicit 20, 40, 60, 80 and 100% of the *W*_max_ previously determined (during a preliminary test day). The incremental tests were counterbalanced across the participants and were undertaken in a ∼22°C environment with fan cooling. Details of the experimental protocols used in study 1 have been reported (Calbet *et al*. 2007; Boushel *et al*. 2011; Helge *et al*. 2011; Boushel *et al*. 2014).

Study 2 formed part of an investigation on the effects of increasing heart rate with right atrial pacing on cardiovascular capacity in healthy humans (Munch *et al*. 2014). The present study 2 focuses on the relationship between central and exercising limb blood temperature and blood flow during incremental leg exercise and quantification of heat transfer from the exercising leg to the body core. In this context, and similar to the incremental exercise protocols in study 1, the endurance‐trained cyclists completed an incremental leg‐cycling test to volitional exhaustion with the work rate being increased every 2 min to elicit 25, 40, 55, 70, 85 and 100% of *W*_max_ (416 ± 16 W). Exercise was performed at ∼22°C with a fan directed against the participant's back. In both studies, the participants’ *W*_max_ and ${\dot{V}}_{O_{2}\max}$ values were assessed during preliminary testing and familiarization visits.

To undertake the lower and upper limb exercise in study 1, a frame was built to host two Monark cycle ergometers (Monark AB839E, Vansbro, Sweden) on top of each other. The lower ergometer was used for leg cycling, while the top cycle ergometer was adapted for arm cranking. Prior to exercise, the height of the upper ergometer was adjusted such that the position of the trunk was vertical and the glenohumeral joint axis at the height of the cranking axis of the arm cycle. The first exercise bout consisted of 90 min of leg and arm exercise. Prior to exercise and at 30, 60 and 90 min of exercise, blood was sampled simultaneously from the arterial and the two venous lines. This was immediately followed by measurements of blood flow in the leg and arm. During incremental leg cycling and arm cranking, blood samples and limb blood flows were measured after 45 s of each exercise stage while blood temperatures were recorded continuously. In study 2, the participants performed incremental leg cycling on an ergometer (Excalibur Sport; Lode, Groningen, The Netherlands). A similar time line was used for blood sampling and leg blood‐flow measurements during the incremental exercise bouts in the two studies (Fig. 1).

### Experimental preparation

In study 1, participants reported to the laboratory at 08.00 h, and then rested supine while catheters where inserted under local anaesthesia (2% lidocaine; Pharmacy Department, Herlev Hospital, Herlev, Denmark). A 20‐gauge catheter (Arrow, Reading, PA, USA) was inserted using the Seldinger technique into the right femoral artery, 2–5 cm below the inguinal ligament, and advanced in the proximal direction. This catheter was connected to a pressure transducer set (T1002009; Baxter, Unterschleissheim, Germany), which allowed for blood sampling and the assessment of arterial pressure with a transducer positioned at the height of the fourth intercostal space (T100209A; Baxter, Unterschleissheim, Germany). A second 20‐gauge catheter was then inserted in the right femoral vein, 2 cm from the inguinal ligament, and advanced in the distal direction for femoral venous blood sampling. In the same femoral vein, a third catheter with side‐holes (Radiopack TFE; Cook, Bjaerverskov, Denmark) was inserted and advanced ∼5 cm proximal to the inguinal ligament for injection of ice‐cold saline. A thermistor (model 94‐030‐2.5F T.D. Probe; Edwards Edslab, Baxter, Irvine, CA, USA) was inserted through the latter catheter for measuring blood flow of the leg (LBF) with the constant‐infusion thermodilution technique (Andersen & Saltin, 1985). To obtain blood samples and measure arm blood flow, a Swan‐Ganz catheter (model 132F5; Edwards Edslab) was inserted into an antecubital vein and advanced to the subclavian vein, with the tip positioned in the midclavicular line (the final position was verified by X‐ray). The tip lumen was used for blood sampling and was connected to a transducer (T100209A; Baxter) to measure the pressure in the subclavian vein. The other lumen was used for infusion of iced saline solution for blood‐flow measurements. Infusate temperature was measured with a flow‐through chamber (model 93‐505; Edslab) connected to the venous catheters. The blood and infusion temperatures and arterial pressure were recorded online via a data‐acquisition system (MacLab 16/s; ADInstruments, Sydney, NSW, Australia) and simultaneously displayed on a monitor (Dialogue 2000; Danica, Copenhagen, Denmark).

In study 2, the participants reported to the laboratory at 08.00 h after a light breakfast. After 30 min of supine rest, catheters were inserted under local anaesthesia (2% lidocaine). One 20‐gauge catheter was inserted in the left radial artery for blood sampling and pressure measurements. An 18‐gauge catheter was then inserted in the femoral vein, 2–3 cm from the inguinal ligament, and advanced in the retrograde direction for blood sampling. A catheter with side‐holes in the tip was also inserted in the same femoral vein in the anterograde direction, and a thermistor was advanced through the catheter for determination of leg blood flow via constant‐infusion thermodilution. Next, a Swan‐Ganz catheter (131HF7; Edwards Life Sciences, Irvine, CA, USA) was inserted under pressure guidance in the pulmonary artery via a left antecubital vein. The catheter allowed for measurement of central mixed venous blood temperature and oxygen variables.

### Experimental and analytical procedures

In both studies, limb blood flow was measured with constant‐infusion thermodilution (Andersen & Saltin, 1985). Briefly, ice‐cold saline was infused simultaneously through both the femoral and subclavian veins in study 1 and through the femoral vein in study 2 at flow rates sufficient to decrease blood temperature at the thermistor by 0.5–1.0°C. Infusate and blood temperatures were measured during saline infusion (Harvard pump; Harvard Apparatus, Millis, MA, USA) via thermistors connected to the data‐acquisition system (MacLab 16/s; ADInstruments). Infusate temperature was measured with a thermistor set in a flow‐through chamber (model 93‐505; Edslab) connected to the venous catheter. At rest, saline infusions were continued for at least 60 s, while during exercise infusions lasted 15–20 s, until blood temperature had stabilized. Blood flow was calculated using a thermobalance equation (Andersen & Saltin, 1985; González‐Alonso *et al*. 2000). Infusate temperature was corrected according to the methodology reported in Calbet *et al*. 2015 (study 1) and González‐Alonso *et al*. 2000 (study 2).

Blood temperature in the femoral and subclavian veins was recorded with the thermistors used for the determination of blood flow, whereas *T*_B_ in the pulmonary artery was measured with a thermistor in the catheter. Blood temperature data reported here represent the average steady‐state values in each of the experimental conditions before blood samples and flows were obtained. Blood samples were repeatedly collected during the prolonged and incremental exercise protocols and rapidly analysed for haemoglobin (Hb), oxygen saturation ($S_{O_{2}}$ *S*_O2_), oxygen tension ($P_{O_{2}})$ and other blood gas variables, which are not the focus of this report. Blood gas variables were corrected for the corresponding *T*_B_ values. The blood oxygen content was calculated from the saturation ($S_{O_{2}})$ and [Hb], i.e. [(1.34 [Hb] × $S_{O_{2}}$) + (0.003 × $P_{O_{2}}$)]. Leg ${\dot{V}}_{O_{2}}$ was the product of LBF and arterial‐to‐femoral vein O_2_ content differences and arm ${\dot{V}}_{O_{2}}$ the product of arm blood flow (ABF) and arterial‐to‐subclavian O_2_ content difference. Leg and arm vascular conductance were calculated as LBF and ABF divided by mean arterial pressure.

In each stage of exercise in study 2, blood samples were drawn simultaneously from the radial and pulmonary arteries and femoral vein at rest and after 1.5 min of exercise at each work rate. Pulmonary ${\dot{V}}_{O_{2}}$ was measured online (Quark CPET system; Cosmed, Rome, Italy). Leg blood flow was measured immediately before and after blood sampling. Blood temperature was recorded via a data‐acquisition system (PowerLab 16/30; ADInstruments, Bella Vista, NSW, Australia), and cardiac output was calculated using the Fick equation (cardiac output = ${\dot{V}}_{O_{2}}$/arterial − venous O_2_ difference). Heat transfer from the exercising legs to the body core was estimated by multiplying the pulmonary artery‐to‐femoral venous *T*_B_ differences by LBF and the specific heat of blood (3.6 kJ l^−1^ °C^−1^), assuming that *T*_B_ in the pulmonary artery reflects that of the femoral artery. Leg aerobic heat production was calculated by subtracting the heat equivalent of power output (developed by a single leg, i.e. one‐half of power output during two‐legged cycling) from the heat equivalent of leg aerobic energy turnover. The latter was estimated as leg ${\dot{V}}_{O_{2}}$ multiplied by the heat equivalent of oxygen (21 kJ l^−1^).

Plasma [ATP] was determined at rest and during steady‐state leg‐ and arm‐cycling exercise (study 1) with the luciferin–luciferase technique (Lundin, 2000), using a luminometer with two automatic injectors (ORION Microplate Luminometer; Berthold Detection System GmbH, Pforzheim, Germany). Blood samples (2.7 ml) for determination of [ATP] were obtained using syringes containing EDTA (S‐monovette, 2.7 ml KE; Sarstedt, Nümbrecht, Germany) and centrifuged immediately for 30 s at 15,493 g (4°C; Sigma 1‐15 K, Osterode am Harz, Germany). Plasma was then pipetted into prechilled tubes, frozen in dry ice and stored for later analysis. Plasma [ATP] was measured in duplicate at room temperature (∼20°C) using an ATP Kit (ATP Kit SL 144‐041; BioTherma AB, Dalarö, Sweden) with an internal ATP standard procedure. Plasma [Hb] was also analysed spectrophotometrically to determine whether haemolysis had occurred during the handling of the samples (Cripps, 1968). In addition, plasma noradrenaline and adrenaline concentrations were determined at rest and at 30, 60, 75 and 90 min of steady‐state leg‐ and arm‐cycling exercise (study 1) using high‐performance liquid chromatography with electrochemical detection (Hallman *et al*. 1978).

### Statistical analysis

A two‐way repeated‐measures ANOVA was performed to test significance between treatments. Following a significant *F* test, pair‐wise differences were identified using the Bonferroni *post hoc* procedure. Linear regression was used for analysis of the relationship amongst blood flow, blood temperature, plasma [ATP] and ${\dot{V}}_{O_{2}}.$ The significance level was set at *P < *0.05, and data are presented as mean values ± SEM unless otherwise stated. All analyses were carried out using MAC SPSS Statistics (version 20; IBM Corporation, Armonk, NY, USA).

## Results

### Blood temperature, blood flow and ${\dot{V}}_{O_{2}}$ in exercising and non‐exercising limbs

**Figure 2:**
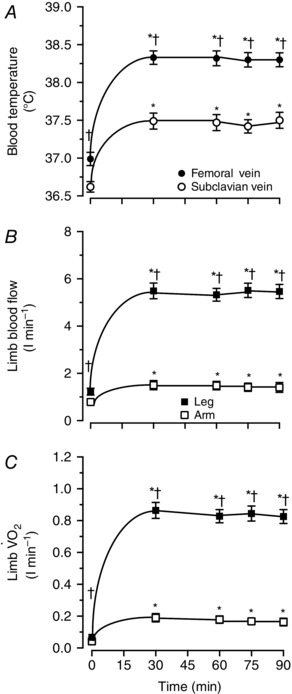
**Blood temperature, limb perfusion and oxygen uptake (** ${\dot{V}}_{O_{2}}$ **) during combined leg and arm exercise** Blood temperature, limb blood flow and ${\dot{V}}_{O_{2}}$ in the leg and arm during 90 min of moderate‐intensity constant‐load leg and arm cycling. *A*, blood temperature in the femoral and subclavian veins. *B*, limb blood flow in the leg and the arm. *C*, limb ${\dot{V}}_{O_{2}}$ in the leg and the arm. Data are means ± SEM for 16 subjects. *Higher than 0 min baseline, *P* < 0.05. ^†^Higher than arm values, *P* < 0.05.

At the onset of prolonged constant‐load leg and arm exercise (study 1), limb *T*_B_, blood flow and ${\dot{V}}_{O_{2}}$ increased in both legs and arms (Fig. 2). However, all these variables reached higher absolute ‘steady‐state’ values in the legs than in the arms (i.e. +0.8 ± 0.1°C, +4.0 ± 0.4 l min^−1^ and +0.7 ± 0.1 l min^−1^ for *T*_B_, limb blood flow and limb ${\dot{V}}_{O_{2}},$ respectively; all *P *< 0.05). The difference in ‘steady‐state’ limb ${\dot{V}}_{O_{2}}$ disappeared when the data were normalized per unit of work (i.e. 16 ± 1 *versus* 16 ± 1 ml min^−1^ W^−1^ in the exercising leg and the exercising arm, respectively) or per rate of aerobic metabolism as reported below.

**Figure 3:**
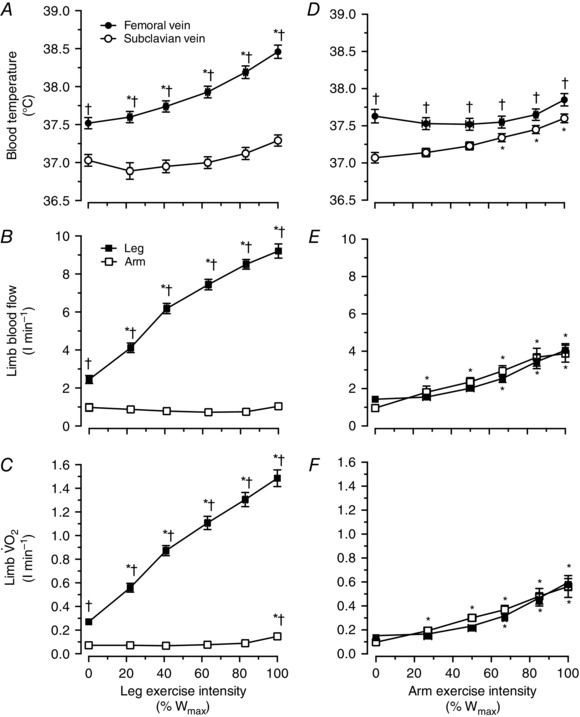
**Blood temperature, limb perfusion and oxygen uptake during incremental leg or arm exercise** Blood temperature, limb blood flow and ${\dot{V}}_{O_{2}}$ in the leg and arm during incremental leg cycling and incremental arm cranking. *A*, blood temperature in the femoral and subclavian veins during incremental leg exercise. *B*, limb blood flow in the leg and the arm during incremental leg exercise. *C*, limb ${\dot{V}}_{O_{2}}$ in the leg and the arm during incremental leg exercise. *D*, blood temperature in the femoral and subclavian veins during incremental arm exercise. *E*, limb blood flow in the leg and the arm during incremental arm exercise. *C*, limb ${\dot{V}}_{O_{2}}$ in the leg and the arm during incremental arm exercise. Data are means ± SEM for 16 subjects. *Different from control, *P* < 0.05. ^†^Higher than arm values, *P* < 0.05.

During incremental leg cycling, LBF gradually increased in parallel with elevations in *T*_B_ and limb ${\dot{V}}_{O_{2}},$ whereas ABF, *T*_B_ and ${\dot{V}}_{O_{2}}$ remained unchanged or increased at exhaustion (Fig. 3). Thus, *T*_B_, LBF and ${\dot{V}}_{O_{2}}$ were all elevated at rest and at each stage of exercise in the exercising leg compared with the arms (all *P < *0.05). During incremental arm cranking, both ABF and LBF increased, as did limb ${\dot{V}}_{O_{2}}$ (Fig. 3 *B* and *C*). No significant differences between limbs in blood flow or ${\dot{V}}_{O_{2}}$ were observed during incremental arm‐cranking exercise (Fig. 3 *E* and *C*). However, *T*_B_ was higher in the legs than in the arms at rest and during exercise (Fig. 3 *D*).

**Figure 4:**
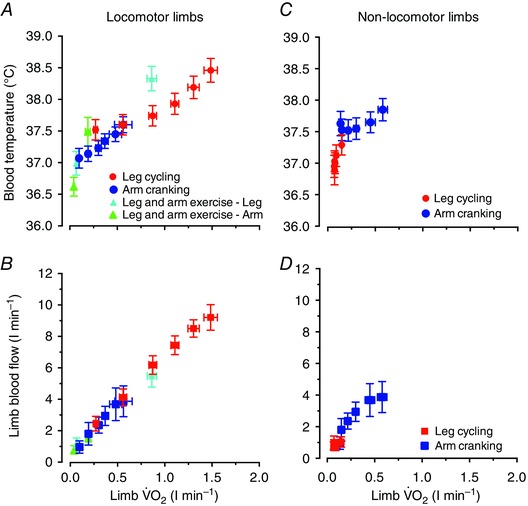
**Blood temperature, limb perfusion and aerobic metabolism** Relationships between blood temperature *versus* limb ${\dot{V}}_{O_{2}}$ (*A*) and limb blood flow *versus* limb ${\dot{V}}_{O_{2}}$ (*B*) at baseline and during incremental and prolonged leg and/or arm exercise (locomotor limbs graphs). The same relationships are also depicted for the non‐locomotor limbs during incremental leg cycling (i.e. the arms) and arm cranking (i.e. the legs; *C* and *D*). Data are the means ± 95% confidence interval for 15–16 participants. The relationship between *T*_B_ and limb ${\dot{V}}_{O_{2}}$, established using linear regression analysis, was strong (*A*), particularly when focusing on the incremental leg‐cycling and arm‐cranking data (*y* = 0.90*x* + 37.04; *r* ^2^ = 0.95; *P < *0.05). A correlation was also observed between limb perfusion and ${\dot{V}}_{O_{2}}$ when including all data points from all the interventions (*y* = 5.97*x* + 0.64; *r* ^2^ = 0.99; *P < *0.05; *B*). In the non‐locomotor limbs, the relationships between *T*_B_ and limb ${\dot{V}}_{O_{2}}$ (*C*) and limb blood flow and limb ${\dot{V}}_{O_{2}}$ (*D*) were also significant, although not as strong as in the locomotor limbs.

The relationships between *T*_B_ and limb perfusion *versus* limb ${\dot{V}}_{O_{2}}$ in the locomotor and non‐locomotor limbs are depicted in Fig. 4 *A–D*. Figure 4 *A* and *B* also includes the baseline and exercise data from the incremental and prolonged exercise conditions. In the locomotor limbs, there was a correlation between *T*_B_ and limb ${\dot{V}}_{O_{2}}$, particularly when focusing on the incremental leg‐cycling and arm‐cranking data (*y* = 0.90*x* + 37.04; *r* ^2^ = 0.95; *P < *0.05; Fig. 4 *A*). The relationship was still significant when including the data from all the conditions (*y* = 1.01*x* + 36.99; *r* ^2^ = 0.83; *P < *0.05). The relationship between limb perfusion and ${\dot{V}}_{O_{2}}$ was also significant when including in the analysis the data from all the interventions (*y* = 5.97*x* + 0.64; *r* ^2^ = 0.99; *P < *0.05; Fig. 4 *B*). The relationships between *T*_B_ and limb perfusion *versus* limb ${\dot{V}}_{O_{2}}$ were also significant in the non‐locomotor limbs.

### Plasma ATP and catecholamines in the exercising legs and arms

At rest, plasma ATP and noradrenaline in the femoral and subclavian veins were not statistically different (i.e. 1.2 ± 0.2 *versus* 1.0 ± 0.1 μmol l^−1^ and 1.5 ± 0.2 *versus* 1.5 ± 0.2 nmol l^−1^, respectively; *P > *0.05). These two variables increased in both vessels during leg and arm exercise; however, after 30 min the plasma ATP and noradrenaline were higher in the femoral than the subclavian vein (1.8 ± 0.3 *versus* 1.4 ± 0.2 μmol l^−1^ and 4.5 ± 0.3 *versus* 3.8 ± 0.3 nmol l^−1^, respectively; *P < *0.05). In contrast, plasma adrenaline was lower in the femoral than in the subclavian vein both at rest and during exercise (rest, 0.18 ± 0.03 *versus* 0.31 ± 0.04 and exercise, 0.46 ± 0.07 *versus* 0.63 ± 0.08 nmol l^−1^, respectively; *P < *0.05).

### Relationships among limb vascular conductance, blood temperature, plasma ATP, blood oxyhaemoglobin and metabolism

**Figure 5:**
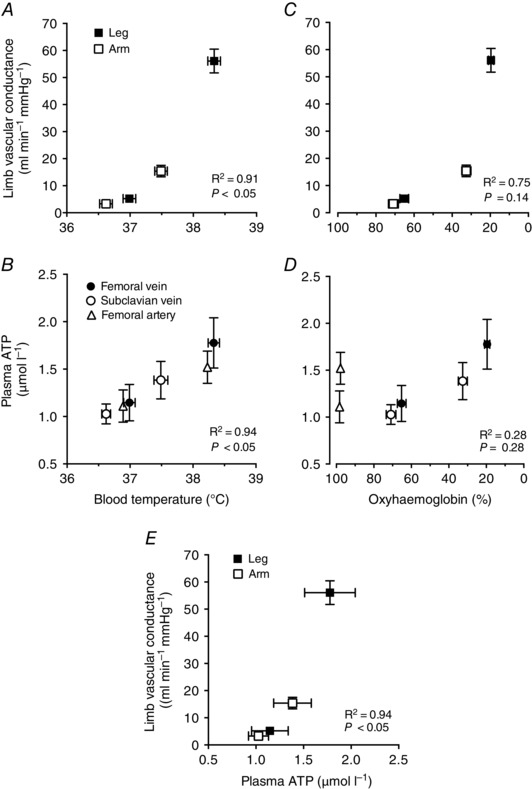
**Limb vascular conductance, plasma ATP, blood oxyhaemoglobin and blood temperature** *A–E*, relationships between resting and steady‐state leg and arm exercise values for limb vascular conductance and plasma [ATP] plotted against blood temperature (*T*_B_) and blood oxyhaemoglobin as well as limb vascular conductance plotted against plasma [ATP]. The relationships amongst these variables were established using linear regression analysis. Data are means ± SEM for 15–16 participants. Note the significant correlation between limb vascular conductance *versus T*_B_ (*r* ^2^ = 0.91; *P < *0.05; *A*) and [ATP] (*r* ^2^ = 0.94; *P < *0.05; *E*) and between [ATP] *versus T*_B_ (*r* ^2^ = 0.99; *P < *0.05; *B*). In contrast, the relationship between limb vascular conductance and venous and arterial blood oxyhaemoglobin was not significant (*r* ^2^ = 0.75; *P = *0.14; *C*). The increase in venous [ATP] was correlated with reductions in venous blood oxyhaemoglobin (*r* ^2^ = 0.91; *P < *0.05). However, [ATP] was also elevated during exercise in the arterial blood despite an unchanged arterial oxyhaemoglobin, making the overall correlational analysis of arterial and venous samples insignificant (*D*).

In the combined leg and arm exercise protocol of study 1, leg and arm vascular conductance, and thus the local vasodilatation in the legs and arms during exercise compared with rest, was correlated with elevations in *T*_B_ (*r* ^2^ = 0.91; *P < *0.05) and [ATP] (*r* ^2^ = 0.94; *P < *0.05; Fig. 5 *A* and *E*), but not with venous blood oxyhaemoglobin (*r* ^2^ = 0.75; *P = *0.14; Fig. 5 *C*). In turn, venous [ATP] levels in the legs and arms at rest and during prolonged leg and arm exercise were correlated with venous limb *T*_B_ (*r* ^2^ = 0.99; *P < *0.05; Fig. 5 *B*) and venous blood oxyhaemoglobin (*r* ^2^ = 0.91; *P < *0.05; Fig. 5 *D*). Femoral artery [ATP] also increased during prolonged leg and arm exercise compared with rest (1.5 ± 0.2 *versus* 1.1 ± 0.2 μmol l^−1^; *P* < 0.05), despite arterial oxyhaemoglobin remaining at ∼ 98% (Fig. 5 *D*). When including both the arterial and the venous samples in the analysis, [ATP] and *T*_B_ remained highly correlated (*r* ^2^ = 0.94; *P < *0.05), whereas [ATP] and oxyhaemoglobin did not (*r* ^2^ = 0.28; *P = *0.28). Femoral artery *T*_B_ was assumed to be 0.1°C lower than femoral venous *T*_B_ based on the difference in *T*_B_ seen in study 2 between the femoral vein and the pulmonary artery.

### Blood temperature, blood flow and ${\dot{V}}_{O_{2}}$ in the exercising limbs and the central circulation and heat transfer in the exercising limbs

**Figure 6:**
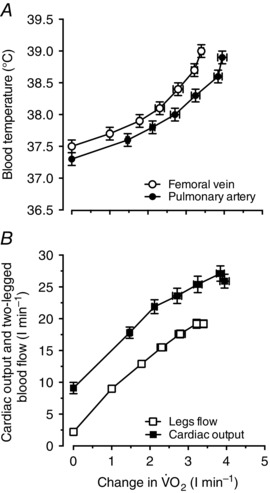
**Central and peripheral blood temperature and perfusion responses as a function of oxygen uptake during incremental leg exercise** Relationship between blood temperature and perfusion *versus* aerobic metabolism during incremental leg cycling to volitional exhaustion. *A*, blood temperature in the femoral vein and pulmonary artery during incremental leg exercise plotted against increases in leg and systemic ${\dot{V}}_{O_{2}}$ from baseline. *B*, cardiac output and two‐legged blood flow during incremental leg exercise plotted against increases in leg and systemic ${\dot{V}}_{O_{2}}$ from baseline. Data are means ± SEM for 12 subjects. Note that mean *T*_B_ values in the femoral vein and the pulmonary artery during the different stages of exercise were within ∼0.1°C.

During incremental exercise in the trained endurance cyclists in study 2, *T*_B_ in the femoral vein and pulmonary artery increased at the same rate from initial values of 37.5 ± 0.1 and 37.3 ± 0.1 to 39.0 ± 0.1 and 38.9 ± 0.1°C, respectively, at the time of exhaustion. The averages between vessel *T*_B_ were within ∼0.1°C (Fig. 6 *A*). The LBF and leg ${\dot{V}}_{O_{2}}$ responses during incremental leg cycling were mirrored by similar cardiac output and systemic ${\dot{V}}_{O_{2}}$ dynamics (Fig. 6 *B*).

**Figure 7:**
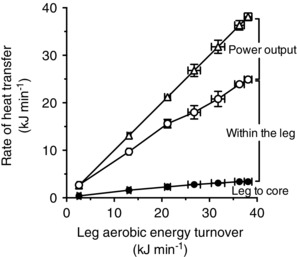
**Heat transfer in the exercising limb** Relationship between leg aerobic energy turnover and the rate of heat transfer from the leg to the body core, within the leg and the heat equivalent of power output. Data are means ± SEM for 12 subjects. Note that heat transfer within the leg represented ∼60% of the leg aerobic energy turnover during each exercise stage. Heat transfer within the leg will be higher when accounting for the anaerobic heat production during moderate and high exercise intensities, which could not be quantified in this study.

Leg aerobic energy turnover increased progressively from 2.6 ± 0.4 kJ min^−1^ at baseline to 38.1 ± 0.8 kJ min^−1^ at maximal exercise, whereas the corresponding estimated heat transfer from the leg to the body core increased from 0.4 ± 0.1 to 3.4 ± 0.1 kJ min^−1^ and the heat equivalent of power output developed by a single leg rose from nil to 13.5 ± 0.5 kJ min^−1^ (Fig. 7). Likewise, the leg aerobic heat production rose from 2.6 ± 0.4 kJ min^−1^ at baseline to 24.5 ± 1.1 kJ min^−1^ at maximal exercise, whereas the corresponding heat transfer within the leg (i.e. the aerobic heat production not accounted for by heat transfer to the body core) increased from 2.3 ± 0.4 to 21.4 ± 0.6 kJ min^−1^. Heat transfer within the leg represented ∼60% of the leg aerobic energy turnover during each exercise stage.

## Discussion

The principal finding of the present investigation was a close coupling amongst perfusion, *T*_B_ and aerobic metabolism, both in the distinct metabolically active exercising limbs during combined leg and arm exercise and in the exercising and non‐exercising limbs during separate incremental leg‐cycling or arm‐cranking exercise. The magnitude of vasodilatation in the leg and arm vascular beds, as reflected by changes in limb vascular conductance during prolonged leg and arm exercise compared with rest, was closely correlated with elevations in limb *T*_B_, plasma ATP and ${\dot{V}}_{O_{2}}$ (all *r* ^2^ = 0.91–0.99; *P < *0.05). The finding in study 2 that increases in femoral venous *T*_B_ and leg perfusion during strenuous incremental leg cycling were mirrored by similar pulmonary artery *T*_B_ and cardiac output dynamics, together with the observation in study 1 of unchanged subclavian venous *T*_B_ and arm perfusion, suggest that processes in active limbs largely determine central *T*_B_ and blood‐flow responses to exercise. Together, these findings suggest that mechanisms sensitive to temperature and metabolic rate are involved in the regulation of limb perfusion, possibly through signalling pathways that control the intravascular concentration of ATP.

### Temperature, metabolism and limb perfusion

This integrative human physiology investigation characterized the *T*_B_, perfusion and ${\dot{V}}_{O_{2}}$ responses of the human limbs to different whole‐body exercise modalities and activity levels. An important observation was the relationships amongst *T*_B_, perfusion and aerobic metabolism during dynamic exercise with the lower and upper limbs alone, or combined lower and upper limb exercise. In agreement with the literature, the rate of rise in blood flow in either exercising limb in relationship to the elevation in limb ${\dot{V}}_{O_{2}}$ was 5.7–6.4 l min^−1^ l^−1^ during separate incremental leg and arm exercise (Andersen & Saltin, 1985; Knight *et al*. 1992; Volianitis & Secher, 2002; Mortensen *et al*. 2005, 2008). Likewise, the rate of rise in limb perfusion in relationship to the increase in local *T*_B_ was 5.5–7.2 l min^−1^ °C^−1^. The latter range includes values in the non‐exercising yet metabolically active legs during arm cranking and in the exercising legs of the trained cyclists, who exhibited greater increases in temperature, haemodynamic and exercise capacity responses than the volunteers of study 1. The link between *T*_B_, perfusion and metabolism was further supported by the observation that *T*_B_, ABF and ${\dot{V}}_{O_{2}}$ remained essentially unchanged at ∼37°C, ∼0.9 l min^−1^ and ∼0.09 l min^−1^ in the non‐exercising arms during strenuous incremental leg cycling, when these variables increased progressively to ∼38.5°C, ∼9.2 l min^−1^ and ∼1.5 l min^−1^ in the exercising legs. Interestingly, the reciprocal phenomenon did not occur during incremental arm cranking because the leg muscles became active in maintaining an upright body position required for dynamic arm exercise. Consequently, leg perfusion increased to the same peak value as in the arms (∼4 l min^−1^), accompanied by the same elevation in aerobic metabolism and an increased *T*_B_ starting from the early stages of exercise (Fig. 3). Taken together, these observations are consistent with the concept that limb *T*_B_ and perfusion depend upon local aerobic metabolism and heat production.

The finding that venous *T*_B_ and perfusion during incremental leg cycling remained stable in the non‐exercising arms despite profound increases in *T*_B_ and perfusion in the exercising legs has implications for the understanding of regional temperature and blood‐flow regulation during exercise. Adding information on the central circulatory responses to incremental leg cycling, we found similar elevations in pulmonary artery and femoral venous *T*_B_, in agreement with the literature showing large increases in core and brain temperatures (Saltin *et al*. 1966; Nybo *et al*. 2002; Kenny *et al*. 2003; Trangmar *et al*. 2014) but smaller changes in *T*_B_ and lower *T*_B_ and muscle temperature in the resting arm (González‐Alonso *et al*. 1999; Jay *et al*. 2007). The local thermal stimuli modulating thermoregulatory and blood‐flow responses during exercise are therefore weaker in the non‐exercising limbs. During prolonged exercise, 60–65% of metabolic heat production in the exercising limbs is transferred to the neighbouring tissues and the overlying skin (González‐Alonso *et al*. 1999). In support of this phenomenon, we estimated that the rate of heat transfer from the exercising legs to the body core at each stage of incremental exercise in trained individuals accounted for ∼16% of the rate of leg aerobic heat production (Fig. 7). Leg anaerobic heat production was not estimated, but its contribution to total metabolic heat production during maximal exercise is substantial (González‐Alonso *et al*. 2000; Bangsbo *et al*. 2001; Krustrup *et al*. 2003). The fractional rate of metabolic heat production transferred to the body core is therefore small. These findings identify the active limbs as major sites of temperature and blood‐flow regulation during exercise.

The increases in *T*_B_ in the large vessels draining blood from the legs and arms (i.e. femoral and subclavian veins) during exercise following a short warm‐up period reflect the increase in contracting skeletal muscle tissue and blood temperature (Sproule & Archer, 1959; Saltin & Hermansen, 1966), owing to elevations in heat production during muscle contraction (González‐Alonso *et al*. 2000), and the simultaneous rapid thermal equilibration between tissues and vessels (He *et al*. 2002). Our data reveal that this measure of mixed venous *T*_B_ is sensitive to haemodynamic and metabolic changes in the limb tissues during dynamic exercise. That said, heterogeneity in the thermal, haemodynamic and metabolic responses ought to occur within the different skeletal muscle groups and limb tissues of the upper and lower extremities (Kalliokoski *et al*. 2001; Vogiatzis *et al*. 2015), because the present dynamic exercise modalities engage only a fraction of the total limb muscle mass (Ray & Dudley, 1998; Richardson *et al*. 1998). Also, ${\dot{V}}_{O_{2}}$ and perfusion are different among muscle groups during dynamic exercise (Vogiatzis *et al*. 2015). Yet the observations that local variations in ${\dot{V}}_{O_{2}}$‐to‐perfusion ratio are minimal suggest that the local blood flow is closely matched to the regional metabolic rate of the working muscles (Vogiatzis *et al*. 2015). In this light, differences in *T*_B_, perfusion and ${\dot{V}}_{O_{2}}$ between lower and upper limbs during combined leg and arm exercise became small or disappeared when expressing the data per unit of work. Furthermore, the global limb blood flow‐to‐${\dot{V}}_{O_{2}}$ relationship during separate and combined leg and arm exercise was tight (Fig. 4 *B*). Collectively, these observations indicate that the regulation of the local thermal, haemodynamic and metabolic responses of the upper and lower limbs to dynamic exercise involves common regulatory mechanisms.

### Temperature‐ and metabolism‐sensitive mechanisms for control of limb tissue perfusion

A large number of local and central mechanisms have been proposed to regulate active skeletal muscle blood flow, including metabolic, thermal, myogenic, mechanical, humoral and neural signalling pathways (Rowell, 1993, 2004; Saltin *et al*. 1998; Laughlin *et al*. 2012; Joyner & Casey, 2015). Regulation of skin blood flow in response to thermal stimuli also involves local and reflex neural mechanisms (Johnson *et al*. 2014). The tight relationships amongst limb perfusion, *T*_B_ and ${\dot{V}}_{O_{2}}$ support the idea that thermal and metabolic stimuli contribute to exercising limb hyperaemia. In keeping with a role of thermal stimuli, increases in local blood and muscle temperature are associated with similar elevations in limb perfusion during isolated leg or whole‐body heat stress (Pearson *et al*. 2011; Heinonen *et al*. 2011; Chiesa *et al*. 2015) and during combined heat stress with one‐legged knee‐extensor exercise (Pearson *et al*. 2011; Chiesa *et al*. 2015). However, the resulting leg hyperaemia amounts to only ∼0.6–1.1 l min^−1^ (Pearson *et al*. 2011; Chiesa *et al*. 2015) compared with the up to ∼9 l min^−1^ leg hyperaemia during leg cycling, or even the ∼4 l min^−1^ arm hyperaemia observed during maximal arm cranking. Thus, metabolic hyperthermia *per se* plays only a modest role in exercise hyperaemia.

Erythrocyte‐derived ATP is a metabolic and thermal sensing mechanism thought to contribute to local vascular control in limb tissues during dynamic exercise and thermal hyperaemia (Ellsworth *et al*. 1995; González‐Alonso *et al*. 2002; Kalsi & González‐Alonso, 2012). ATP is an attractive mediating signal for skeletal muscle blood‐flow regulation, not only because it can act as a potent vasodilator capable of increasing leg perfusion by up to ∼8 l min^−1^ (Duff *et al*. 1954; González‐Alonso *et al*. 2002, 2008), but also because of its sympatholytic properties in the human limb circulation (Rosenmeier *et al*. 2004; Kirby *et al*. 2008). To gain insight into this mechanism, we measured plasma ATP, blood oxygenation and plasma noradrenaline in the subclavian and femoral veins and in the brachial artery at rest and during combined steady‐state leg and arm exercise. Plasma [ATP] was higher in the femoral than in the subclavian vein during exercise in association with higher perfusion. A close association was therefore established between the increases in [ATP] and leg and arm vasodilatation (Fig. 5 *E*). The increased vasodilatation in the legs occurred despite a higher plasma noradrenaline concentration, indicating that the high ATP and/or other vasoactive substance overrode the effect of enhanced vasoconstrictor activity in the leg (Rosenmeier *et al*. 2004; Kirby *et al*. 2008). Comparison of the strength of the relationships between [ATP] *versus T*_B_ and [ATP] *versus* oxyhaemoglobin provides information about two potential mechanisms leading to increases in intravascular [ATP]. In venous blood, plasma [ATP] was tightly associated with increases in *T*_B_ and reduction in oxyhaemoglobin (Fig. 6 *B* and *D*), suggesting that erythrocyte ATP release has been stimulated by activation of thermal and oxygen‐sensitive pathways (Ellsworth *et al*. 1995; Kalsi & González‐Alonso, 2012). However, when including data from both arterial and venous samples, the increase in [ATP] was significantly related only to *T*_B_. Erythrocytes from arterial and venous samples, but not plasma or serum, release ATP in a temperature‐dependent manner (Kalsi & González‐Alonso, 2012). Both *in vivo* and *in vitro* data therefore support the hypothesis that hyperthermia in conjunction with metabolic stimuli contributes to regulation of perfusion to active limbs by stimulating ATP release from erythrocytes.

In conclusion, the present findings in healthy human subjects reveal a close coupling between perfusion and blood temperature in the exercising and non‐exercising limbs during separate leg cycling and arm cranking and in the different metabolically active limbs during combined leg and arm exercise. During constant‐load upper and lower limb exercise, limb vascular conductance, and thus the magnitude of vasodilatation in the vasculature of the legs and arms during exercise compared with rest, was related to elevations in *T*_B_, plasma ATP and limb ${\dot{V}}_{O_{2}}$. These data are consistent with the hypothesis that temperature‐ and oxygen‐sensitive intravascular ATP release contributes to regulation of limb perfusion in humans. Yet the cause‐and‐effect relationships amongst blood temperature, oxygenation, ATP and flow remain to be elucidated.

## Additional information

### Competing interests

None declared.

### Author contributions

J.G.‐A., J.A.L.C., R.B., J.W.H., H.S. and G.v.H. were involved in the conception and design of study 1 with respect to the present report, whereas J.G.‐A., S.P.M. and N.H.S. conceived and designed study 2. J.G.‐A, J.A.L.C., R.B., J.W.H., T.M‐A., H.S. and G.v.H. were involved in data collection, analysis and/or interpretation of data of study 1, whereas J.G.A., S.P.M. and N.H.S. did so in study 2 with respect to the present focus. J.G.‐A. wrote the article, which was critically revised by the co‐authors. All authors approved the final version of the manuscript.

### Funding

The studies were conducted at the Copenhagen Muscle Research Centre. Study 1 was supported by the Danish National Research Foundation (504‐14) and the John and Birthe Meyer Foundation. Study 2 was supported by Team Denmark.
